# Palliative care‐based arguments against assisted dying

**DOI:** 10.1111/bioe.13352

**Published:** 2024-10-03

**Authors:** Ben Colburn

**Affiliations:** ^1^ Department of Philosophy University of Glasgow Glasgow Scotland

**Keywords:** assisted dying, criminal law, ethics, euthanasia, palliative care

## Abstract

Opponents of legalised assisted dying often assert that palliative care is worse in countries where assisted dying has been legalised, and imply that legalised assisted dying makes palliative care worse. This study considers five versions of this claim: that it is difficulty to access expert palliative care in countries where assisted dying has been legalised, that those countries rank low in their quality of end‐of‐life care; that legalising assisted dying doesn't expand patient choice in respect of palliative care; that growth in palliative care services has stalled in countries where assisted dying has been legalised; and that legalised assisted dying impedes the growth of palliative care or causes it to decline. In each case, it concludes that neither argumentation nor evidence supports these claims.

## 1 INTRODUCTION

Opponents of assisted dying often assert that palliative care is worse in countries where assisted dying has been legalised and imply that legalised assisted dying makes palliative care worse or that assisted dying is legalised only where palliative care is underdeveloped. This study shows that the evidence supports neither the assertion nor the implications. It does not seek to show that palliative care cannot or will not deteriorate if legalised assisted dying is introduced; that depends on broader questions of government support and funding for palliative care, which should remain an urgent priority whether assisted dying is legalised or not. Nevertheless the study does show that we should reject the claims of those who seek to oppose assisted dying by appealing to the importance of palliative care.

Opponents of legalised assisted dying make a cluster of claims about its relationship with palliative care:

1. It is difficult to access expert palliative care in countries where assisted dying has been legalised;
2. Countries where assisted dying has been legalised rank low in their quality of end‐of‐life care;
3. Legalising assisted dying doesn't expand patient choice in respect of palliative care;
4. Growth in palliative care services has stalled in countries where assisted dying has been legalised;
5. Legalised assisted dying impedes the growth of palliative care or causes it to decline.

Before moving on to evaluating these claims against the evidence, it is worth mentioning two problematic features of the *way* that these claims have been advanced. Opposition to assisted dying on these grounds is characterised by *firehosing* and by *insinuation in place of argumentation*. The apparent force of these worries depends to some extent on these discreditable features, rather than on the merits of the worries themselves.

A great many recent publications in which these claims appear are authored by the same people, a comparatively small group of active and retired clinicians, academics, and senior figures at UK campaigning organisations like the Christian Medical Fellowship, Care Not Killing, and its offshoot Our Duty of Care. They appear not in research studies but rather in non peer‐reviewed letters to the editors of research journals and broadsheet newspapers.^1^ This proliferation of letters is reminiscent of ‘firehosing’ tactics^2^: it generates the appearance of widely shared and mutually supportive findings. That appearance is specious. Putting these sources together, we see not mutual and independent reinforcement but rather the repetition of the same assertions by a small group of the same people. This point should not be overstated, because there are some others besides this cluster of writers who have made some of the same claims,^3^ but the appearance of a widespread expert belief that assisted dying makes palliative care worse is an illusion.

A second problem is that—as the rest of this paper will show—these authors depend on insinuation rather than explicit argumentation to convey the idea that palliative care is worsened by assisted dying. Oftentimes there is a significant gap between what is explicitly said on the page and the implicit message that assisted dying should not be legalised. The plausibility of these claims *as reasons to oppose assisted dying* in fact depends on this gap. If the missing reasoning were made explicit and shared candidly, it would be transparently weak. The case against legalisation is not at all supported by the evidence being adduced, even where that evidence is reported accurately (which is not always true). There is, in other ways, something covert about the way these claims are advanced: their persuasive power depends upon key elements remaining hidden.^4^

The rest of this study evaluates the claims made about the relationship between assisted dying and palliative care, concentrating for the most part on the sources their proponents themselves cite as supporting their claims, on the basis that these are presumably the best evidence available. In each case, my conclusion is that the claims are false, misleading, unsupported by evidence, or some combination of these.

## 2 IS IT DIFFICULT TO ACCESS EXPERT PALLIATIVE CARE IN COUNTRIES WHERE ASSISTED DYING HAS BEEN LEGALISED?

In a letter of 2022 Claud Regnard and co‐authors say that ‘many papers … show the difficulties in accessing expert palliative care in countries where AD [assisted dying] has been legalised’.^5^ They cite four sources to support this claim.^6^ Inspection of those sources shows that, for different reasons, none of them supports Regnard and his co‐authors' assertion.

Arias‐Casais et al. studied trends in specialised palliative care service provision between 2005 and 2019, counting specialist services in 51 European countries in 2005, 2012, and 2019. Its main conclusions were that service provision generally increased during the study period, but remained below the EAPC recommendation of 0.5 services per 100,000 inhabitants,^7^ particularly in central/eastern European and lower income countries.^8^

I discuss Arias‐Casais et al.'s findings about the *trends* in palliative care provision below, when I ask whether growth in palliative care services has stalled in countries where assisted dying has been legalised. On the question of whether access to palliative care is *difficult* in those countries, Arias‐Casais et al. considered which countries met the EAPC recommendation. Their results show that all four countries with legalised assisted dying either met this benchmark (Luxembourg and Switzerland) or came close to achieving it (Belgium and the Netherlands, with only three others).^9^

So, Arias‐Casais et al. didn't demonstrate ‘difficulties in accessing expert palliative care in countries where AD has been legalised’, as Regnard and others have claimed. In fact, their study reveals *more* such services in those countries than is generally the case elsewhere.

Jordan et al. wrote a systematic review and meta‐analysis, drawing on 169 studies between 2‐2013 and 2018 in 23 countries, determining the weighted median duration adults in each country spent in palliative care between initiation and death. Researchers found a wide range, from 6 days in Australia to 69 days in Canada, with their main finding being ‘a negative correlation between duration of palliative care and country level of human development’.^10^

Jordan et al.'s conclusions do not ‘show the difficulties in accessing expert palliative care in countries where AD has been legalised’.^11^ There are two key problems here. First, it isn't clear what the connection is between the study's focus (weighted median duration of palliative care) and Regnard and co‐authors' claims (about accessibility of expert palliative care). Jordan et al. emphasise the importance of ‘timely initiation’, but don't say that the explanation for a short weighted median duration is lack of access and explicitly acknowledge that a short median duration of palliative care might indicate that a country continues to offer curative rather than palliative care for longer in terminal illness.^12^

Second, Jordan et al. do not discuss assisted dying at all. They draw no conclusions about the effects of legalised assisted dying. Their study included three countries with legalised assisted dying: Belgium, the Netherlands, and Canada (where it was legalised in 2016).^13^ Of those, Canada had the longest weighted median duration in the entire study (68.88 days), the Netherlands a duration of 36.00 days, nearly twice the global figure of 18.91 days, and Belgium a duration of 17.95, just below the global figure.^14^ These data are scattered across the range, and show nothing about the effects of assisted dying.

So, Jordan et al. 2020 do not demonstrate that there is ‘difficulty in accessing expert palliative care in countries where AD has been legalised’.^15^ It draws no conclusions about this. And if the intended effect was to insinuate further that there is difficulty in accessing expert palliative care in those countries *because* AD has been legalised, Jordan et al.'s results undermine, rather than support, that interpretation.

Mitchell et al. studied hospital deaths in Australia between July 2015 and June 2016, and showed that ‘recognition of death is predominantly within the last 48 h of life’, which ‘minimises timely palliative care’.^16^ The study period preceded the legalisation of assisted dying (which first came into effect in Victoria in 2019) by several years. So, it is disingenuous to present this study as showing ‘the difficulties in accessing expert palliative care in countries where AD has been legalised’^17^—assisted dying hadn't been legalised in Australia at the time—or as supporting the implication that patients had difficulties accessing expert palliative care *because* AD has been legalised.

Munro et al. studied 84 patients at The Ottawa Hospital in Ontario who requested Medical Assistance in Dying (MAiD) between February 2016 and June 2017, comparing the level of palliative care involvement before and after those requests. It found that 59.5% of patients had palliative care involvement of some sort before their MAiD requests, that 38% were offered palliative care after requesting MAiD, and 46.4% still had involvement with the hospital palliative care team after their requests. The study concludes that ‘[t]here is still inadequate provision of palliative care for those requesting MAiD’.^18^

Munro et al.'s central finding is incongruent with other evidence, for example Health Canada's report that 77.6% of all MAiD recipients across the country received palliative care before their requests, and 87.5% had it ‘accessible if needed’.^19^ Commentators have doubted the wisdom of drawing general conclusions from Munro et al.'s local findings.^20^

Even granting this central finding, Munro et al. make other claims whose argumentative or evidential basis is unclear. No specific argument is given that their results reveal ‘inadequate’ palliative care provision. It seems just one of several possible ways to interpret the drop in active involvement seen after MAiD requests and is surprising given their own findings (which indicate that at least 88% of patients were offered palliative care before or after their MAiD requests and that a large majority probably received it at some stage in the process).^21^ Perhaps the thought is that everyone *should* receive palliative care, and hence that anything less than a 100% participation rate counts as ‘inadequate’. But once we make that (worryingly paternalistic) implicit assumption explicit, we can see that it not supported by the evidence. Elsewhere, key claims—for example, ‘Concerns have been raised that those without adequate access to palliative care might have a higher symptom burden and therefore a higher likelihood to seek MAiD as a means to address their suffering’^22^—are unattributed or unsupported. In general, there is a failure to distinguish research findings from evaluative commentary.

So, Munro et al.'s research findings—as opposed to their dubiously related evaluative commentary—don't show that Canadians in general have difficulty accessing expert palliative care if they want it, as the Health Canada report shows.^23^ It's not even clear they show that about the small sample of patients studied. So, the explicit claim that it is one of ‘many papers [that] show the difficulties in accessing expert palliative care in countries where AD has been legalised’.^24^ is false. Moreover, Munro et al. give no reason to accept the unstated implication that the patients in their study, much less Canada more generally, had difficulties in accessing expert palliative care *because* AD has been legalised.

To summarise, the first claim under consideration is that palliative care is difficult to access in countries where assisted dying has been legalised. The evidence shows that this is false, and also refutes the further implication that palliative care is difficult to access *because* assisted dying has been legalised.

## 3 DO COUNTRIES WHERE ASSISTED DYING HAS BEEN LEGALISED RANK LOW IN THEIR QUALITY OF END‐OF‐LIFE CARE?

Regnard and co‐authors claim that ‘AD legislatures rank low in their quality of end‐of‐life care compared with the non‐AD countries of UK and Ireland’.^25^ They cite a study by Finkelstein et al. in support of this point.^26^

Finkelstein et al. conducted and analysed a cross‐country survey of 181 experts from 81 countries, carried out between May and August 2021, assessing the quality of death and dying in their own countries against thirteen key indicators (including ‘managed pain and discomfort’, ‘clean and safe space’, ‘kind treatment’, ‘clear and timely information’, ‘preferred place of death’, and ‘contact with family’).^27^ Countries were scored and ranked. The United Kingdom and Ireland came at the top of the ranking, with scores of 93.1 and 92.9, respectively, out of a notional maximum of 100.^28^

Finkelstein et al. do not explicitly discuss assisted dying in their study. Of the 81 countries surveyed, four allowed assisted dying at the time of the survey: Switzerland (score of 87.6, rank 13), Canada (score of 81.2, rank 22), Belgium (score of 80.7, rank 26), and Colombia (score of 71.9, rank 42).^29^ The Netherlands and Luxembourg don't appear in their list.

The United Kingdom and Ireland come top of the ranking, so Regnard and co‐authors' claim that AD legislatures ‘rank low … compared with the non‐AD countries of UK and Ireland’ is true.^30^ However, focusing just on countries' ordinal position—where they sit in the ranking—is selective and misleading. Looking also at countries' cardinal scores—how their services were graded against the criteria—puts those ordinal rankings into context. All four countries with legalised assisted dying had cardinal scores in the top quartile (taking 93.1, the UK's score, as the maximum achieved), and all were graded C or above by Finkelstein et al., which means they were ranked higher than the United States, inter alia. Regnard and co‐authors' implication that these findings support their anti‐assisted dying conclusion depends on this choice to focus only on countries' ordinal rankings rather than their more revealing cardinal scores. The latter indicate that palliative care provision is comparatively strong in countries with legalised assisted dying.

Regnard and co‐authors also claim that ‘all AD legislatures have dropped in their rankings since 2015 in the Quality of Death Index’.^31^ The 2015 ranking they refer to—the Economist Intelligence Unit's 2015 *Quality of Death Index*—is not directly comparable to Finkelstein et al.'s study, because the rankings had different methodologies, sufficiently different that Finkelstein et al. explicitly discuss limitations in the earlier study's methodology.^32^ So, a direct comparison of countries' positions in these rankings is of scant use. Moreover, even if we think the comparison meaningful, Regnard and co‐authors' claims about it are inaccurate. Two of the four assisted dying countries had higher ordinal ranks and cardinal scores in the later ranking: Colombia, whose ordinal rank rose from 68 to 42, and whose cardinal score rose from 26.7 to 71.9; and Switzerland, whose rank rose from 15 to 13, and whose score rose from 76.1 to 87.6. Canada's rank dropped from 11 to 22, but its score rose from 77.8 to 81.2. Only Belgium saw both its rank drop—from 5 to 26—and its cardinal score worsen, although the latter—a drop from 84.5 to 80.3—is much smaller than the ordinal drop would imply.^33^ So, far from ‘all AD legislatures’ dropping in the rankings, the truth is that half of AD legislatures had higher ordinal rankings in the later study, and three‐quarters had higher cardinal scores.

Finally, and echoing my conclusion in the preceding sections, Finkelstein et al.'s own conclusions run counter to the implication that countries with assisted dying are ranked lower than the United Kingdom and Ireland *because* they have legalised assisted dying. Finkelstein et al. identify various factors explaining countries' scores. In respect of some indicators national income is key; in others, the question of how far a country has universal healthcare coverage in general is more important, and in yet others there are more specific factors that had a positive or negative effect. Of the 10 factors that entered negatively, none of those, even obliquely, includes the legal status of assisted dying.^34^

To summarise, the second claim under consideration is that countries with assisted dying rank low in their quality of end‐of‐life care. The evidence shows that this is false, and also refutes the further implication that countries rank low *because* assisted dying has been legalised.

## 4 DOES LEGALISING ASSISTED DYING FAIL TO EXPAND PATIENT CHOICE IN RESPECT OF PALLIATIVE CARE?

Regnard says that ‘claims that legalising assisted dying expands patient choice conflicts with increasing evidence that access to palliative care remains inequitable and inconsistent’.^35^ This somewhat tortuous sentence might encourage a reader to think that legalised assisted dying is ‘increasing’ the inequitability and inconsistency of access to palliative care, and that Regnard has found evidence of it. That is, however, not what Regnard explicitly claims. First, he asserts that *evidence* is increasing of access problems, not that there is evidence of an *increasing* problem: the assertion is just that there are now more studies than there were. Second, Regnard asserts that this evidence conflicts with ‘claims that legalising assisted dying expands patient choice’. That assertion about conflict isn't proved by the evidence; rather, it is Regnard's own interpretation of what the evidence might lead us to conclude about one moral reason amongst others for advocating assisted dying (namely, that it expands patient choice). As above, the gap between what is explicitly asserted and what is merely implied looms large here. Regnard's wording might make a reader think that there is a straightforward and direct evidence base for the authors' critical claims about assisted dying. There is not. A reader might look at exactly the same evidence as Regnard and come to a very difference interpretation.

Let us examine the evidence, and the interpretation, in turn.

As evidence, Regnard cites Munro et al.'s Canadian study,^36^ the problems with which I have already explained in detail above, and a 2018 report by the Canadian Institute for Health Information; the latter bears out his explicit assertion that there are long‐standing problems of equity and consistency in access to palliative care in Canada but nowhere suggested MAiD as a driver of those problems.^37^ The 2023 report by the same institute suggests that things have since improved, notwithstanding the availability of MAiD: ‘more people are receiving some form of palliative care than they were 5 years ago, and more people are dying at home with palliative care support’.^38^ Regnard also cites a Belgian study, but that shows only that ‘measures specifically intended to support palliative *home* care are underused’, with ‘social inequalities in their uptake’^39^; and he cites Mitchell et al.'s Australian study^40^ to claim that there are problems in Australia, despite—as I showed above—that study predating the legalisation of assisted dying by several years.

Moving on to Regnard's interpretation of this scant evidence, none of these studies suggest that legalising assisted dying has *reduced* patient choice in respect of palliative care, even if not enough has been done in other ways to augment it. His interpretation also ignores the way that the mere option of assisted dying by itself augments patient choice, benefiting people whether or not they currently want to take the option.^41^

To summarise, the third claim under consideration is that choice‐based case for legalising assisted dying is undermined because assisted dying doesn't expand patient choice. The evidence shows that this is false.

## 5 HAS GROWTH IN PALLIATIVE CARE SERVICES STALLED IN COUNTRIES WHERE ASSISTED DYING HAS BEEN LEGALISED?

Regnard and co‐authors claim that ‘growth in palliative care services … in Belgium and the Netherlands has stalled since 2012’ and that ‘growth in non‐AD countries in Western Europe has been faster than AD countries’.^42^ The claim is repeated in slightly varied form in a number of places: ‘growth in palliative care services has stalled since 2012 in Belgium and the Netherlands, in contrast to most non‐AD Western European countries’^43^; ‘The reality is that growth in palliative care services is slower in European “assisted dying” countries, with Dutch and Belgian growth static for nearly two decades’^44^; ‘Growth in Dutch and Belgian palliative care services has stalled since 2012, despite increasing demand’^45^; and ‘There was no growth in palliative care services 2012–2019 in Belgium and the Netherlands’.^46^

The sole citation offered to underpin this firehosing is Arias‐Casais et al.'s study, which we have already considered above.^47^ In what follows, I show that this study does not support, but rather confounds, the claims of Regnard and his co‐authors.

Of the four countries with legal assisted dying during the study period, Arias‐Casais et al. found ‘constant increase in service provision’ between 2005 and 2019 in the Netherlands and Switzerland, and an overall increase during that period in Belgium and Luxembourg, though the growth took place only between 2005 and 2012, the same finding as for the United Kingdom.^48^ Given their other finding (discussed above) that all four of these countries had unusually high levels of provision of palliative care services, it is misleading to describe the lack of growth between 2012 and 2019 as ‘stalled’. Rather, the fact that these countries (especially Belgium and Luxembourg) already had comparatively generous provision by 2012 supports Arias‐Casais et al.'s own alternative hypothesis: those countries ‘achieving a saturation of services covering their needs’.^49^ There is no suggestion that legalising assisted dying has anything to do with differences in either growth or absolute levels of provision of palliative care.

As before, we must conclude that Regnard and co‐authors are incorrect to cite Arias‐Casais et al. in support of their assertions. Their study doesn't show that ‘growth in palliative care services … has stalled’ in Belgium and the Netherlands.^50^ In fact it shows *growth* across the study period in both countries. It is misleading to say that ‘growth in non‐AD countries in Western Europe has been faster than AD countries’ given the high starting points in those countries. And the study certainly gives no grounds to conclude that palliative care is undermined by legalising assisted dying.

To summarise, the fourth claim under consideration is that growth in palliative care has stalled in countries where assisted dying has been legalised. The evidence shows that this is false, and also refutes the further implication that growth stalls *because* assisted dying has been legalised.

## 6 DOES LEGALISED ASSISTED DYING IMPEDE THE GROWTH OF PALLIATIVE CARE, OR CAUSE IT TO DECLINE?

The claims considered so far (that palliative care is hard to access in countries where assisted dying has been legalised, that such countries rank low in the quality of end‐of‐life care, that it doesn't support patient choice, or that growth in palliative care services stalls where assisted dying is legal) each face their own problems when faced with even the evidence base cited by their proponents. In addition, a shared pattern has emerged. Even if those explicit claims were true, that wouldn't be enough to show that we should oppose assisted dying. *That* conclusion depends on a further, unspoken, implication that problems with palliative care services in countries where it is legal arise *because* it is legal or that assisted dying is legalised because there are problems with palliative care. These implications—which concern causation, not mere correlation—are crucial for the apparent case against assisted dying. But they have been unsupported by the evidence or argumentation considered so far.

Given the importance of these causal claims, it is interesting that they are seldom made explicitly and directly. Regnard doesn't do so, but rather obliquely asserts that we should ‘question claims that legalising assisted death is compatible with palliative care and does not impede its developments’, targeting papers by Chambaere and Bernheim, and also by Dierickx.^51^ If the implication is that ‘questioning’ the research findings in those papers should lead us to *reject* them, then it's not clear why. Regnard doesn't engage directly with the evidence and argumentation in either paper. Instead he cites a study analysing organisational positions taken by hospices in Oregon on assisted dying which shows that ‘two‐ thirds of hospice programmes did not take part in assisted deaths’,^52^ and a Canadian news story about the Delta Hospice Society, which was seeking to move its hospice to a private site because they didn't want to implement the regional health authority's policy that hospices should provide MAiD.^53^ Neither of those things show that assisted dying impedes the development of palliative care. The Oregon hospices that decline to administer assisted dying remain active in offering palliative care. The Delta Hospice Society did in the end cease operating its hospice,^54^ but the hospice itself continued to operate as a ‘government‐owned and run institution’^55^ and the society has since continued its original activities (of providing direct support to families facing terminal illness and bereavement) and additionally now campaigning against MAiD.^56^

Widening our focus beyond recent correspondence from Regnard and co‐authors, Bernheim et al. allude to ‘the oft‐invoked fear that legally regulated physician‐assisted dying would impede the development of palliative care’,^57^ but its being ‘oft‐invoked’ doesn't translate into its being *oft‐asserted* or *oft‐supported*. Generally, it is just reported. To give two examples—I give more below—a 2003 report for the European Association for Palliative care says that ‘[i]f euthanasia is legalised in any society, then the potential exists for … the underdevelopment or devaluation of palliative care’,^58^ but does not cite evidence for this. Rutherford et al. refer to ‘a feared decline in palliative care resourcing and standards following legalisation’,^59^ but don't indicate who might have such fears, or why. I give further examples in what follows of people referring to these fears. But it is notable that few researchers have been prepared to directly assert and support them.

One exception to that pattern is Jose Pereira, who argues that in countries that have legalised assisted dying ‘rates of palliative care involvement have been decreasing’.^60^ He says this ‘contradicts claims that in Belgium, legalisation has been accompanied by significant improvements in palliative care in the country’.^61^ His argument and evidence for this is confused, however. Pereira cites a study which shows that palliative care involvement *in cases of assisted dying* in Belgium declined from 19% in 2002 to 9% in 2007,^62^ but that doesn't imply anything about involvement in palliative care *in general*, and doesn't contradict other findings that palliative care in general improved over that period.^63^ Pereira's broader claim that in countries without legal assisted dying like the United Kingdom, Australia, Ireland, France, and Spain ‘palliative care has developed more than it has in Belgium and the Netherlands’ has since been refuted by Arias‐Casais et al.,^64^ as we saw above.

So, Pereira's argument is unsound, and confounded by the evidence.

More recently, Worthington et al. claim that ‘evidence from jurisdictions where “assisted dying” is practised reveals a significant impact on clinical practice’.^65^ Besides recounting the news story (mentioned above) about an anti‐MAiD Canadian hospice having to move from land it was leasing from the government^66^ they also cite studies from Canada^67^ and Australia^68^ in support of their claim. These studies discuss some difficulties faced by healthcare professionals in adjusting to the comparatively recent legalisation of assisted dying in those jurisdictions, and also highlight the amount of time and resources dedicated to assisted dying. One of the 23 clinicians interviewed by Mathews et al. in Canada said:

… when a patient is requesting MAID [Medical Assistance in Dying], most of the resources have been sucked up by that one case and it's all everyone's talking about and they're rushing to get stuff done…everyone from admin down to the bedside nurse is focusing on MAID [Medical Assistance in Dying]. And all of the high‐quality palliative care that we do falls by the wayside for the other patients.^69^

Rutherford et al.'s survey of 25 Australian clinicians suggests that ‘coordinating a VAD [voluntary assisted dying] application through to the patient's death equates to about sixty hours of working time’ which is often unremunerated and lacks a wider structure of support save what practitioners can ‘corral from other areas of their medical practice’.^70^

It seems fair to describe these experiences as ‘significant impact’. However, it is a mistake to interpret them as significant impact *from legalisation* on palliative care services. The problem is rather under‐resourcing of the medical system. That is a problem regardless of whether or not assisted dying is legalised, and isn't exacerbated solely by legalisation.

A final point to make about this ‘oft‐invoked fear’^71^ is that it is most commonly mentioned in the context of studies which have concluded that there is no evidence to support it. For example, Gordijn and Janssens alluded to the idea that in a society with expansive assisted dying ‘palliative care would probably not or only insufficiently be developed’ but note that ‘all these predictions are fairly speculative and lacking a sound basis’.^72^ Bernheim et al. found ‘no evidence … that the drive to legalise euthanasia would interfere with the development of palliative care’.^73^ Smets et al. show that only 10% of Belgian physicians agreed that assisted dying was impeding the growth of palliative care.^74^ Chambaere et al. concluded that ‘there is scant evidence of the supposed underdevelopment of palliative care [as a result of legalisation in Belgium and the Netherlands]’.^75^ Chambaere and Bernheim found that ‘[t]he hypothesis that legal regulation of physician‐assisted dying slows development of PC [palliative care] is not supported by the Benelux experience. On the contrary, regulation appears to have promoted the expansion of PC’.^76^ An Australian report considered the concern that introducing assisted dying ‘could stunt the development of the palliative care sector and erose its culture of competent and compassionate care’ and concluded that ‘Evidence to support this concern has not been found’.^77^ Philip et al. allude to the concern ‘that investment in MHD may have an impact upon investment in palliative care—either limiting growth or indeed effectively reducing funding’ and conclude that ‘It is not possible to state if funding to support MHD services has either detracted from or been accompanied by expansion of palliative care services’.^78^

It is ironic that these repeated allusions to the claim that assisted dying impedes the growth of palliative care, or causes it to decline, contribute to the firehosing phenomenon identified in the introduction even when—as here—they occur in the context of research which repeatedly shows that this ‘oft‐invoked fear’ is baseless. In this respect, defenders of assisted dying unintentionally collude with its proponents in making it seem as though this is a widely accepted point. In fact, that is an illusion. Especially in the face of the confounding evidence cited in the preceding paragraph, the repeated invocation of the fear shouldn't be misconstrued as evidence for its being well‐founded.

To summarise, the fifth claim under consideration is that legalised assisted dying impedes the growth of palliative care or causes it to decline. This is the most prominent and common claim we have considered, but the evidence shows that this too is false.

## 7 CONCLUSION

This study has considered each of a cluster of claims made about the effect of legalising assisted dying on palliative care. Evaluating these claims even against the very evidence cited by those advancing it shows that they are groundless: there is no evidence that legalising assisted dying makes palliative care worse. The disconnect, between what is actually shown in the sources cited and the claims made about them, is sometimes astonishing wide. The appearance that this opposition to assisted dying is evidence‐based evaporates as soon as a reader follows up the footnotes.

This is one respect in which this literature falls short of what one might hope from scholarly writing about a weighty question in medical ethics. The other, as we have seen, is that these arguments are frequently characterised by rhetorical tactics of dubious propriety. In particular, it seems that these concerns about palliative care have at least some of their purchase—their ‘oft‐invoked’ status^79^—precisely just because they are talked about so much. This is a situation to which proponents of assisted dying, as well as opponents, have contributed.

The question of whether a country legalises assisted dying is serious, with high stakes. We are ill‐served by a paternalistic approach reliant on the use of rhetoric, misdirection, and insinuation rather than evidence and argumentation. The current study shows that there is no evidence to support the view that legalised assisted dying either causes, or is caused by, poor palliative care. It would be best to treat the question as closed until some new evidence or argument is offered.

## Footnotes

1 For example, Regnard, C. (2021). The impact of assisted dying on hospices and palliative care, *ehopsice*. https://ehospice.com/editorial_posts/the-impact-of-assisted-dying-on-hospices-and-palliative-care/; Regnard, C., Davis, C., Finlay, I., George, R., & Proffitt, A. (2022). Letter to the editor. *The Bulletin of the Royal College of Surgeons of England, 104*, 324–325; Glenny, L., Nyatanga, B., Regnard, C., Bisset, M., Damaso, S., Davis, C., Edwards, F., Fallon, M., George, R., Pollock, J., Proffitt, A., Robinson, V., Thavaraj, A., Twycross, A., Twycross, R., & Wright, G. (2022). Assisted dying. *International Journal of Palliative Nursing*, *28*, 55–58; Regnard, C., & Proffitt, A. (2022, October 7). Letters: Our objections to assisted dying are based on evidence, not religion. *The Guardian*; Proffitt, A., Brooks, D., George, R., Noble, B., & Regnard, C (2022, December 16). Letters. *The Times*; Regnard, C. (2023, October 20). Letters to the editor. *Church Times*.

2 Paul, C., & Matthews, M. (2016). *The Russian “firehose of falsehood” propaganda model: Why it might work and options to counter it*. RAND Corporation. https://www.rand.org/pubs/perspectives/PE198.html

3 For example, Materstvedt, L. J., Clark, D., Ellershaw, J., Førde, R., Boeck Gravgaard, A‐M., Müller‐Busch, H.C., Porta i Sales, J., & Rapin, C‐H. (2003). Euthanasia and physician‐assisted suicide: A view from an EAPC Ethics Task Force. *Palliative Medicine*, *17*, 97–101; Pereira J. (2011). Legalising physician‐assisted dying or assisted suicide: the illusion of safeguards and controls. *Current Oncology*, *18*, 38–45; and Worthington, A., Finlay, I., & Regnard, C. (2023). Assisted dying and medical practice: Questions and considerations for healthcare organisations' *BMJ Supportive & Palliative Care*, *13*, 438–441.

4 Colburn, B. (2015). Authenticity and the third‐person perspective. In G. V. Levey (Ed.) *Authenticity, autonomy and multiculturalism*. Routledge.

5 Glenny, L., et al., op. cit. note 1, p. 55; Regnard (2022), op. cit. note 1; Grey‐Thompson, T., George, R., Proffitt, A., Regnard, C., & Yuill, K. (2023, February 24). Letters to the editor. *The Daily Telegraph*; Regnard (2023), op. cit. note 1.

6 Arias‐Casais, N., López‐Fidalgo, J., Garralda, E., Pons, J. J., Rhee, J. Y., Lukas, R., de Lima, L., & Centeno, C. (2020). Trends analysis of specialized palliative care services in 51 countries of the WHO European region in the last 14 years. *Palliative Medicine*, *34*, 1044–1056; Jordan, R. I., Allsop, M. J., ElMokhallalati, Y., Jackson, C. E., Edwards, H. L. Chapman, E. J., Deliens, L., & Bennett, M. I. (2020). Duration of palliative care before death in international routine practice: A systematic review and meta‐analysis. *BMC Medicine*, *18*, 368; Mitchell I., Lacey, J., Anstey, M., Corbett, C., Douglas, C., Drummond, C., Hensley, M., Mills, A., Scott, C., Slee, J‐A., Weil, J., Scholz, B., Burke, B., & D'Este, C. (2021). Understanding end‐of‐life care in Australian hospitals. *Australian Health Review*, *456*, 540–547; Munro C., Romanova, A., Webber, C., Kekewich, M., Richard, R., & Tanuseputro, P. (2020). Involvement of palliative care in patients requesting medical assistance in dying. *Canadian Family Physician*, *66*, 833–842.

7 Radbruch, L., & Payne, S. (2009). European Association for Palliative Care White Paper on standards and norms for hospice and palliative care in Europe: part 1. Recommendations from the European Association for Palliative Care. *European Journal of Palliative Care*, *206*, 278–289.

8 Arias‐Casais, N., et al., op. cit. note 6.

9 Ibid: 1050.

10 Jordan, R. I., et al., op. cit. note 6, pp. 19–20.

11 Glenny, L., et al., op. cit. note 1, p. 55.

12 Jordan, R. I., et al., op. cit. note 6, p. 19.

13 Data for the United States is aggregated so it is not possible to distinguish between states which do and do not allow assisted dying. Data is also given for Australia and Spain, but the legalisation of assisted dying in those places postdates the study period.

14 Jordan, R. I., et al., op. cit. note 6, table 2.

15 Glenny, L., et al., op. cit. note 1, p. 55.

16 Mitchell, I., et al., op. cit. note 6, p. 541.

17 Glenny, L., et al., op. cit. note 1, p. 55.

18 Munro, C., et al. op. cit. note 6, p. 833.

19 Health Canada. (2022). *Fourth annual report on Medical Assistance in Dying in Canada 2022*. Health Canada, p. 33.

20 For example, Landry, J. T. (2023). Medical assistance in dying (MAiD) in Canada: Why Coelho and colleagues are incorrect to suggest the MAiD framework is in significant distress. *Palliative and Supportive Care*, *21*, 1101–1103, at 1102.

21 Munro, C., et al., op. cit. note 6, table 3. My thanks to an anonymous referee for this point.

22 Munro, C., et al., op cit. note 6, p. 839.

23 Health Canada, op. cit. note 19, p. 33.

24 Glenny, L., et al., op. cit. note 1, p. 55.

25 Ibid.

26 Finkelstein, E. A., Bhadelia, A., Goh, C., Baid, D., Singh, R., Sushma, B., & Connor, S. R. (2022). Cross country comparison of expert assessments of the quality of death and dying 2021. *Journal of Pain and Symptom Management*, *63*, e419–e429.

27 Ibid: e422.

28 Ibid: e425.

29 Ibid: e425. One might argue for further inclusions. Spain (score of 80.3, rank 28) legalised assisted dying on 25 June 2021. In Australia (score of 90.9, rank = 4) assisted dying was legal in Victoria for the whole survey period, and in Western Australia after 1 July 2021; in the United States (score of 71.5, rank 43) assisted dying was legal in nine states and the District of Columbia. Since Finkelstein et al. did not disaggregate responses by state I exclude Australia and the United States here.

30 Glenny, L., et al., op. cit. note 1, p. 55.

31 Ibid.

32 Finkelstein, E. A., et al., op. cit. note 26, p. e420.

33 Economist Intelligence Unit. (2015). *The 2015 Quality of Death Index: Ranking Palliative Care across the World*. http://www.economistinsights.com/healthcare/analysis/quality-death-index-2015; p. 15; Finkelstein, E. A., et al., op cit. note 26, p. e425.

34 Finkelstein, E. A. et al., op cit, note 26, pp. e425–e427.

35 Regnard (2021), op. cit. note 1.

36 Munro, C., et al., op. cit. note 6.

37 Canadian Institute for Health Information. (2018). *Access to Palliative Care in Canada*. Canadian Institute for Health Information.

38 Canadian Institute for Health Information. (2023). *Access to Palliative Care in Canada, 2023*. Canadian Institute for Health Information. My thanks to an anonymous referee for bringing this to my attention.

39 Maetens, A., Beernaert, K., Deliens, L., Gielen, B., & Cohen, J. (2019). Who finds the road to palliative homecare support? A nationwide analysis on the use of supportive measures for palliative home care using linked administrative databases. *PLoS ONE* 14: e0213731. My emphasis.

40 Mitchell, I., et al., op. cit. note 6.

41 Colburn, B. (2020). Autonomy, voluntariness, and assisted dying. *Journal of Medical Ethics*, *45*, 316–319.

42 Glenny, L., et al., op. cit. note 1, p. 55.

43 Regnard (2022), op cit. note 1.

44 Proffitt, A. et al. (2022). op cit. note 6.

45 Regnard & Proffitt, op. cit. note 1.

46 Regnard (2023) op. cit. note 1.

47 Arias‐Casais, N., et al., op. cit. note 6.

48 Ibid: 1048.

49 Ibid: 1055.

50 Glenny, L., et al., op. cit. note 1, p. 55.

51 Regnard (2021) op. cit. note 1; Chambaere K., & Bernheim J. L. (2015). Does legal physician‐assisted dying impede development of palliative care? The Belgian and Benelux experience. *Journal of Medical Ethics*, *41*, 657–660; Dierickx, S., Deliens, L., Cohen, J., & Chambaere, K. (2018). Involvement of palliative care in euthanasia practice in a context of legalised euthanasia: A population‐based mortality follow‐back study. *Palliative Medicine, 32*, 114–122.

52 Campbell, C. S., & Cox J. C. (2012). Hospice‐assisted death? A study of Oregon Hospices on death with dignity. *American Journal of Hospice and Palliative Medicine*, *29*, 227–235.

53 Harding, L. (2021, July 18). Delta Hospice Society in envisions new private MAID free facility. *Western Standard*. https://www.westernstandard.news/news/delta-hospice-society-envisions-new-private-maid-free-facility/article_4f547a0c-6122-5160-b673-a103677fe3ad.html

54 Delta Hospice Society. (2024). *Our history*. https://deltahospicesociety.org/about-us/our-history/

55 Harding, op. cit. note 53.

56 Delta Hospice Society. (2024). *About us*. https://deltahospicesociety.org/about-us/

57 Bernheim J. L., Chambaere, K., Theuns, P., & Deliens, L. (2014). State of palliative care development in European countries with and without legally regulated physician‐assisted dying. *Health Care*, *2*, 10–14, at 11.

58 Materstvedt, L. J., et al., op cit. note 3, p. 99.

59 Rutherford, J., Willmott, J., & White, B. P. (2021). What the doctor would prescribe: Physician experiences of providing voluntary assisted dying in Australia. *Omega*, *87*, 1063–1087: 1076.

60 Pereira, op. cit. note 3.

61 Ibid: e41.

62 Smets, T., Bilsen, J., Cohen, J., Rurup, M. L., Mortier, F., & Deliens, L. (2010). Reporting of euthanasia in medical practice in Flanders, Belgium: Cross sectional analysis of reported and unreported cases. *BMJ*, *341*, c5174.

63 For example, Bernheim, J. L., Deschepper, R., Distelmans, W., Mullie, A., Bilsen, J., & Deliens, L. (2008). Development of palliative care and legalisation of euthanasia: Antagonism or synergy? *BMJ*, 336, 864–867.

64 Arias‐Casais, N., et al., op. cit. note 6.

65 Worthington, A., et al., op. cit. note 3, p. 438.

66 Harding, op. cit. note 53.

67 Mathews, J. J., Hausner, D., Avery, J., Hannon, B., Zimmermann, C., & Al‐Awamer, A. (2021). Impact of medical assistance in dying on palliative care: a qualitative study. *Palliative Medicine*, *35*, 447–454.

68 Rutherford, J., et al., op. cit. note 59.

69 Mathews, J. J., et al., op. cit. note 67, p. 451.

70 Rutherford, J., et al., op. cit. note 59, p. 1070.

71 Bernheim, J. L., et al., op. cit. note 57, p. 11.

72 Gordijn, B., & Janssens, R. (2000). The prevention of euthanasia through palliative care: New developments in the Netherlands. *Patient Education and Counseling*, *41*, 35–46: 43. See also Gordijn, B., & Janssens, R. (2004). Euthanasia and palliative care in the Netherlands: An analysis of the latest developments. *Health Care Analysis*, *12*, 195–207.

73 Bernheim, J. L., et al. (2008), op. cit. note 63, p. 866.

74 Smets, T., Cohen, J., Bilsen, J., Van Wesemael, Y., Rurup, M. L., & Deliens, L. (2011). Attitudes and experiences of Belgian physicians regarding euthanasia practice and the euthanasia law. *Journal of Pain and Symptom Management*, *41*, 580–593.

75 Chambaere, K., Centeno, C., Hernández, E.A., Van Wesemael, Y., Guillén‐Grima, F., Deliens, L., & Payne, S. (2011) *Palliative care development in countries with a euthanasia law*. Commission on Assisted Dying, p. 15.

76 Chambaere & Bernheim, op. cit. note 51, p. 657.

77 Apex Consulting. (2018). *Palliative care Australia: Experience internationally of the legalisation of assisted dying on the palliative care sector*. Apex Consulting, p. 20.

78 Philip, J., Le, B., La Brooy, C., Olver, I., Kerridge, I., & Komesaroff, P. (2023). Voluntary assisted dying/euthanasia: Will this have an impact on cancer care in future years? *Current Treatment Options in Oncology*, 24, 1351–1364: 1357.

79 Bernheim, J. L., et al., op cit. note 57, p. 11.

